# Severe fever with thrombocytopenia syndrome complicated with subdural hematoma: A rare case and literature review

**DOI:** 10.1002/jgf2.273

**Published:** 2019-09-09

**Authors:** Takeshi Endo, Norio Yamamoto, Shinichiro Inoue, Tsutomu Yoshikane, Naoki Fujisawa, Toshihiro Imada, Shuzo Hattori

**Affiliations:** ^1^ Department of Internal Medicine Unnan City Hospital Unnan‐City Japan; ^2^ Department of Orthopedics Unnan City Hospital Unnan‐City Japan; ^3^ Department of Neuropsychiatry Okayama University Graduate School of Medicine, Dentistry, and Pharmaceutical Science Okayama-City Japan; ^4^ Department of Neurosurgery Shimane University Faculty of Medicine Izumo-City Japan; ^5^ Division of Virology Shimane Prefectural Institute of Public Health and Environmental Science Matsue‐City Japan; ^6^ Department of General Medicine Shimane Prefectural Central Hospital Izumo‐city Japan

**Keywords:** consciousness disturbance, intracranial hemorrhage, severe fever with thrombocytopenia syndrome, subdural hematoma

## Abstract

A 79‐year‐old woman presented with fever and general malaise. Examination revealed hepatic injury, thrombocytopenia, skin lesions, and regional lymphadenopathy; severe fever with thrombocytopenia syndrome (SFTS) was diagnosed using polymerase chain reaction. The patient developed impaired consciousness that worsened after 4 days. Magnetic resonance imaging of the head revealed a subdural hematoma in the occipital region with an uncertain onset time. As SFTS rarely causes intracranial hemorrhage, the associated risk factors are unknown. Clinicians may overlook potential intracranial hemorrhage in stuporous SFTS patients.

## Abstract 2

We described a rare severe fever with thrombocytopenia (SFTS) case with subdural hematoma. As in this case, there is a possibility that a small amount of intracranial hemorrhage combined with SFTS may be overlooked.

## Graphical abstract

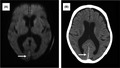

## 1 INTRODUCTION

Severe fever with thrombocytopenia syndrome (SFTS) is a tick‐borne infection caused by the SFTS virus. To date, 397 cases of SFTS have been reported in Japan, with a mortality rate of 18.9%.^1^

The main symptoms of SFTS are fever, liver injury, and thrombocytopenia.^2^ However, association of intracranial hemorrhage with SFTS is not well known. Herein, we describe a rare case of SFTS with subdural hematoma.

## 2 CASE REPORT

A 79‐year‐old woman from a rural area of Shimane Prefecture, Japan, developed a fever that worsened until she could neither drink water nor stand 3 days later. She presented at our hospital on day 4 with a fatigued appearance. Her annual health checkup showed no particular abnormalities; she did not use medications. She sometimes encountered deer on her farm. Two years earlier, she had sustained a subdural hematoma in the right occipital region after falling and bruising her head, which disappeared after 2 months without surgery. Her Glasgow Coma Scale (GCS) score was 15/15; following were her vital signs: temperature, 39.3°C; pulse rate, 60 beats/minute (regular); blood pressure, 131/69 mm Hg; peripheral capillary oxygen saturation, 95%; and respiratory rate, 22 breaths/minute.

Physical examination revealed decreased skin turgor and an asymptomatic reddish pustule on her right flank. No signs of inflammation (eg, pain, lymph node swelling) were observed. Initial laboratory investigation revealed leukopenia and thrombocytopenia (white blood cell count, 2400/μL; platelet count, 73 000/μL). Her liver function test results were abnormal (aspartate transaminase [AST], 80 IU/L; alanine transaminase [ALT], 29 IU/L; creatine kinase [CK], 364 IU/L), but her coagulation profile (prothrombin time ratio, 90.9%; activated partial thromboplastin time, 32.4 seconds) and kidney function (creatinine and urea levels) were normal. Her Epstein‐Barr virus immunoglobulin M titer was not elevated. Plain chest and abdominal computed tomography (CT) showed no abnormal findings.

On day 5, we suspected *Rickettsia* sp. infection and initiated a minocycline hydrochloride drip (200 mg/day). On day 6, bone marrow aspiration cytology revealed an increased proportion of macrophages (13%) with phagocytosed platelets. She had a high fever lasting > 1 week, progressively decreasing red blood cell and platelet counts, and hemophagocytic cells that accounted for ≥3% of her bone marrow nucleated cells. Based on these criteria,^3^ we diagnosed hemophagocytic syndrome; intravenous dexamethasone (13.2 mg/day) was initiated. Re‐examination of plain chest and abdominal CT scans revealed swelling of the right external iliac and superficial inguinal lymph nodes (right flank region) without splenomegaly. As the skin lesion was asymptomatic, the hospital dermatologist attributed the rash to a tick bite.

Although her fever resolved after day 7, she became stuporous^4^ and mostly slept except during meal times (GCS score, 14 points = E3V5M6). On day 8, serological *Rickettsia* examination yielded negative findings. On day 9, an SFTS polymerase chain reaction test yielded positive results (viral RNA load, 1.4 × 10^6^ copies/mL);^5^ thus, we diagnosed SFTS. She received blood platelets (10 units) following a decrease in platelet count to 17 000/μL. We also diagnosed atrial fibrillation, which did not disappear until day 10; thus, we initiated oral rivaroxaban (10 mg/day).

**Figure 1:**
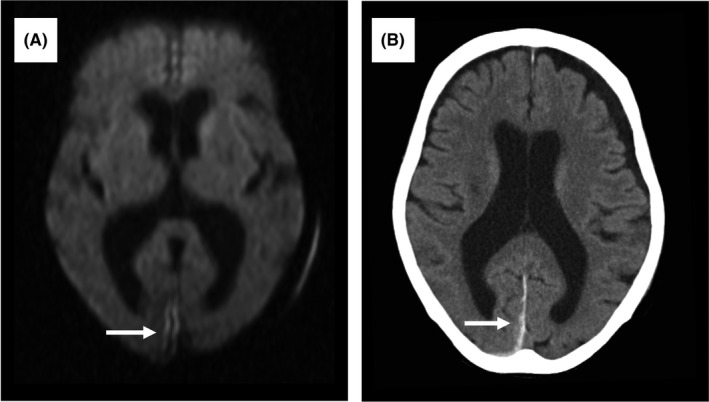
Imaging features of the right occipital region. A, Diffusion‐weighted magnetic resonance imaging on day 11 revealing an area of hyperintensity. B, Noncontrast computed tomography on day 19 revealing an area of hyperdensity

On day 11, she became deeply stuporous (GCS, 11 points = E3V3M5).^4^ She responded affirmatively to all questions but determining whether she was in pain was difficult. She had no other visible symptoms (eg, quadriplegia, facial paralysis, anisocoria). Head magnetic resonance imaging showed a small subdural hematoma in the same right occipital site where a hematoma had developed 2 years earlier (Figure 1A). Her atrial fibrillation disappeared on day 11, and rivaroxaban was stopped.

**Figure 2:**
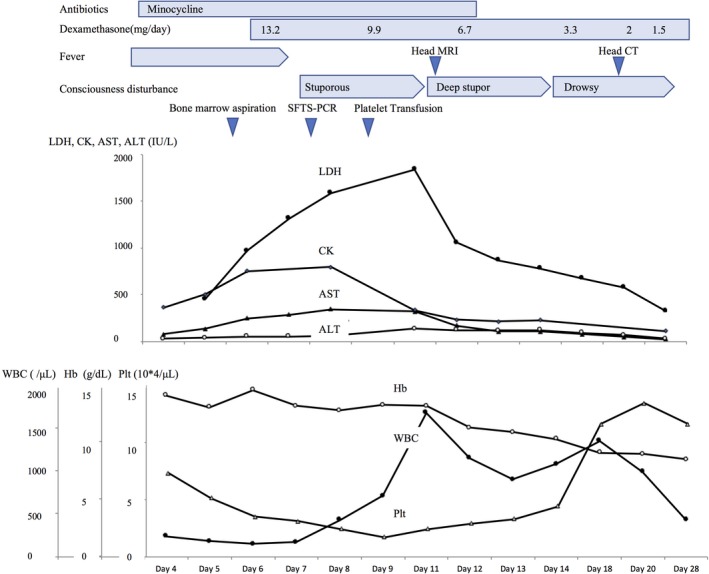
Clinical course of the case, including features, laboratory data, and drug administration. ALT, alanine transaminase; AST, aspartate transaminase; CK, creatine kinase; CT, computed tomography; Hb, hemoglobin; LDH, lactate dehydrogenase; MRI, magnetic resonance imaging; Plt, platelet count; WBC, white blood cell count

After day 12, her AST, ALT, and lactate dehydrogenase (LDH) levels began to decrease. On day 14, her consciousness improved to drowsiness (GCS, 14 points = E4V4M6);^4^ on day 19, plain head CT confirmed no increase in the subdural hematoma (Figure 1B). She was discharged without sequelae on day 47. Figure 2 shows her clinical course.

## 3 DISCUSSION

Central nervous system disorders, multiple organ failure, elevated serum AST/ALT/LDH/CK levels, high viral loads, and hemorrhagic symptoms have been reported as factors associated with fatal SFTS.^6^, ^7^ Previous reports have described gastrointestinal, pulmonary, and gingival hemorrhages and subcutaneous petechiae but not intracranial hemorrhage.^6^, ^7^ Only 3 cases of SFTS with intracranial hemorrhage have been identified, all of which were fatal. According to Jaeseung et al,^8^ only two of 35 patients (6%) with SFTS with intracranial hemorrhage were hospitalized. Yoo et al^9^ reported a case of SFTS with sudden‐onset subdural hematoma in the right frontotemporoparietal region, causing death postsurgery. Given the rarity of SFTS‐related intracranial hemorrhage, the common onset time, risk, and mortality of the hemorrhage could not be determined.

Our patient's age and platelet count at the time of subdural hematoma onset were similar to those described in the case reported by Yoo et al;^9^ platelet transfusions were performed in both cases. In our case, the subdural hematoma was small; however, Yoo et al reported dilated pupils and a large fatal hematoma with a midline shift on CT.

In our case, we supposed that the subdural hematoma did not expand because it involved venous microbleeding rather than arterial bleeding.^10^ However, even if the subdural hemorrhage is not arterial, hematoma expansion remains possible. The steps required to prevent expansion of SFTS‐related subdural hematomas remain unclear, and SFTS‐associated intracranial hemorrhage may be fatal. Thus, early hematoma detection and preparation for emergency surgery are important.

Although our patient had subdural hematoma history, predicting SFTS‐related intracranial hemorrhage was difficult; her subdural hematoma may have developed several days prior to its discovery. Identifying the hematoma was difficult owing to her consciousness disturbance and small size of the subdural hematoma. SFTS is often associated with disturbed consciousness of unknown etiology;^2^ such consciousness disorders hinder observation of subjective symptoms, such as headache and minor paralysis. Furthermore, because the subdural hematoma in this case was small, no objective findings indicative of intracranial hemorrhage (like pupil dilation) were observed. This suggests that intracranial hemorrhages may be overlooked in comatose patients with SFTS. Further studies are needed to determine the timing of hemorrhage development and risk of bleeding for preventing fatalities associated with SFTS‐related intracranial hemorrhage.

## CONFLICTS OF INTEREST

The authors have stated explicitly that there are no conflicts of interest in connection with this article.

## ETHICS APPROVAL

Informed consent was obtained to publish this case report.
